# Highly precise optical detection of mass destruction nerve agents based on photonic crystal fibers

**DOI:** 10.1038/s41598-025-20186-4

**Published:** 2025-09-18

**Authors:** Mohamed Z. Elabdein, Nazmi A. Mohammed, El-Sayed M. El-Rabaie, Omar E. Khedr

**Affiliations:** 1 Electronics and Communications Engineering Department, Alexandria Higher Institute for Engineering and Technology, Alexandria, Egypt; 2 Department of Electronics and Communications Engineering, Faculty of Electronic Engineering, Menoufia University, Menouf, 32952 Egypt; 3 Electronics and Communication Engineering Department, Faculty of Engineering, Sinai University, Arish, Egypt; 4 College of Engineering, Ahlia University, Manama, Kingdom of Bahrain

**Keywords:** PCF, Chemical hazards, Relative sensitivity, Confinement loss, Terahertz, Chemical engineering, Electrical and electronic engineering

## Abstract

Nerve agents such as Sarin, Soman, and Tabun are among the most lethal chemical warfare agents, classified as mass destruction agents due to their extreme toxicity and rapid disruption of the nervous system. These highly volatile and easily dispersible compounds can be deployed in warfare or acts of terrorism, causing fatal respiratory failure, seizures, and irreversible nerve damage even at minimal exposure. The urgency of detecting these agents with high precision is critical for global security and counterterrorism efforts. To address this challenge, a highly sensitive photonic crystal fiber (PCF) sensor with an elliptical cladding and circular core (E-PCF) is designed for the rapid and accurate detection of nerve agents in the terahertz (THz) spectrum. The sensor employs circular air holes in the vestibule region to enhance light-matter interaction, optimizing detection through key performance metrics such as relative sensitivity, effective material loss, and confinement loss. Using two materials, such as silica glass and Zeonex as background materials, the proposed sensor demonstrates exceptional sensitivity and minimal loss. Numerical analysis within the 1.6–3.6 THz range reveals outstanding performance for Sarin (99.6% relative sensitivity, 3 × 10⁻^13^ dB/m confinement loss), Soman (98.8% relative sensitivity, 1.1 × 10⁻¹² dB/m loss), and Tabun (98% relative sensitivity, 7.6 × 10⁻^11^ dB/m loss). With its exceptional optical properties, silica glass ensures highly reliable detection, making the proposed sensor a powerful tool for counterterrorism efforts, environmental monitoring, industrial hazard detection, and military defense. This innovative PCF-based sensing technology marks a major breakthrough in chemical warfare agent detection, providing a fast, precise, and efficient solution for identifying highly toxic substances that pose severe threats to public safety and national security.

## Introduction

Sarin, Soman, and Tabun are highly toxic nerve agents classified as weapons of mass destruction due to their extreme lethality and rapid disruption of the nervous system^1,2^. Even minimal exposure to these agents can lead to severe health consequences, including respiratory failure, seizures, and unfortunately death at the end^3^. Their potential use in warfare or acts of terrorism represents a significant threat to global security and public safety. Additionally, their tenacity in the surroundings and the difficulties related to their detection underscore the need for advanced monitoring and mitigation strategies^4^.

Various techniques are employed to detect toxic chemicals, ranging from conventional laboratory methods to advanced sensor technologies^5,6^. Chemical compounds can be efficiently separated and analyzed using chromatographic methods like gas chromatography (GC)^7^ and liquid chromatography (LC)^8^ are often coupled with mass spectrometry (MS) for precise identification. Spectroscopic techniques, among them ultraviolet-visible (UV-Vis) along with infrared (IR) spectroscopy, identify specific chemical signatures^6^. Additionally, electrochemical sensors and biosensors enable real-time monitoring by detecting molecular-level interactions. Emerging technologies, such as nanomaterial-based sensors and AI-integrated detection systems, enhance sensitivity and speed, improving efficiency in environmental, industrial, and security applications^9,10^.

Optical sensors have a significant part in various fields due to their high sensitivity, fast response time, and non-invasive nature^11–13^. These sensors recognize variations in light properties, such as absorption, fluorescence, or scattering, to identify specific substances, making them valuable for environmental monitoring, medical diagnostics, and industrial applications^14–16^. In chemical and biological sensing, optical sensors can detect toxins, pollutants, or biomarkers at extremely low concentrations, enabling early detection of hazardous substances^17–19^. Their ability to provide real-time and remote monitoring enhances safety in critical areas, such as air and water quality assessment, food safety, and biomedical research^20^. Advances in nanotechnology and fiber optics continue to improve their performance, making optical sensors increasingly indispensable in modern detection systems^21^.

An effective solution to the detection and monitoring challenges posed by these hazardous agents is an optical detector^22,23^. Photonic crystal fiber (PCF) sensors are highly effective for rapid and precise detection due to their unique microstructured design^24,25^. By enhancing light-matter interaction through evanescent field effects, they can detect even subtle changes in refractive index, absorption, or fluorescence^26^. This makes them particularly useful for real-time monitoring in biomedical^27,28^, chemical^29^, and environmental applications^30^, offering improved sensitivity and faster response times compared to traditional optical fiber sensors.

Nerve agents are categorized into two main groups: G substances (e.g., sarin, tabun, and soman) and V substances (e.g., VX)^31,32^. G substances primarily enter the body through inhalation, whereas V agents pose a higher risk via skin absorption and aerosol inhalation^33,34^. Chemically similar to organophosphate pesticides, these agents have limited recorded cases of severe human poisoning, though studies on related compounds offer insights into their effects. Exposure can lead to acute symptoms such as respiratory distress, pinpoint pupils, excessive salivation, muscle twitching, seizures, paralysis, and even death^35^. The 1995 Tokyo subway sarin attack resulted in 12 fatalities and nearly 5,000 injuries^36,37^. Although the World Health Organization (WHO) compiles global health data, detailed statistics on nerve agent-related injuries and deaths remain scarce due to their restricted use and data collection challenges^38^.

In summary, continuous advancements are essential to enhance the detection and monitoring of chemical hazards agents, ensuring rapid and accurate identification to mitigate potential threats. Effective sensing technologies must meet key criteria, including high detection sensitivity, minimal signal loss, efficient power utilization, compact design, and fast response time.

This work presents a PCF-based sensor specifically designed for the precise detection of highly toxic nerve agents such as Sarin, Soman, and Tabun. The proposed sensor is designed, simulated, and extensively evaluated, demonstrating exceptional optical performance in the terahertz (THz) range. The performance assessment of photonic crystal fiber (PCF) sensors relies on key metrics that define their efficiency and reliability^39^. Relative sensitivity (*RS*) measures the sensor’s ability to detect small environmental variations, expressed as a shift in resonance wavelength per unit change^40^. Numerical aperture (*NA*) determines light-gathering efficiency, influencing coupling performance^11^. Confinement loss (*L*_*C*_) evaluates light leakage from the core, affecting signal strength and accuracy^11^. Effective material loss (*EML*) accounts for intrinsic absorption and scattering^41^. Birefringence (*B*) quantifies refractive index differences in orthogonal polarization modes, while effective area (*A*_*eff*_) impacts light distribution, nonlinearity, and overall sensor functionality^11^.

The structure of the sensor is outlined in Sect. “Design methodology”, accompanied by a debate of the performance evaluation metrics in Sect. “Analytical mathematics”. Section “Results outcomes, and discussion ” presents the new design’s outcomes, features, and sensing capabilities. The sensor’s capabilities are further validated through a detailed comparison with related photonic chemical sensors in Sect. “Comparative analysis”. Finally, Sect. “Conclusions” offers a conclusion that highlights the performance and features of the introduced design.

## Design methodology

**Fig. 1:**
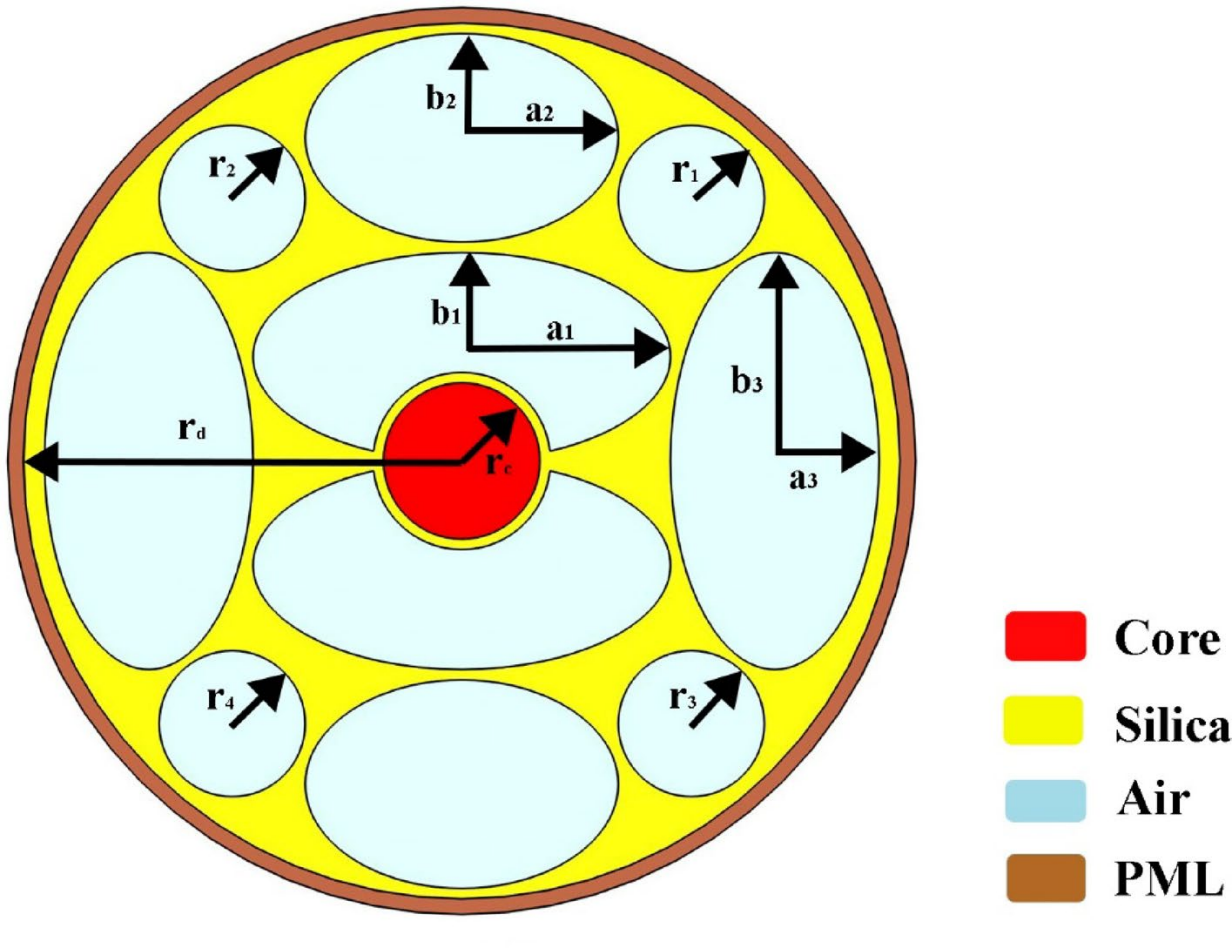
The geometric structure of the resented E-PCF sensor.

The geometric structure of the E-PCF sensor is presented in Fig. 1. The figure represents the cross-sectional design of a photonic crystal fiber (PCF) optimized for use as a chemical sensor. The structure features a central solid core with a radius of (r_d_) = 400 μm, surrounded by a symmetric arrangement of air holes that form two distinct layers. The circular core chosen with a radius (r_c_) = 75 μm. The inner layer consists of four large elliptical air holes evenly distributed around the core, providing strong light confinement, and maximizing interaction with the evanescent field. The two large horizontal elliptical air holes surrounding the core have a width of (a_1_) = 200 μm and height of (b_1_) = 100 μm. While the two large vertical elliptical air holes have a width of (a_2_) = 150 μm, and height of (b_2_) = 100 μm.

The outer layer comprises four smaller circular air holes, symmetrically positioned between the elliptical holes, which contribute to structural stability and additional tunability of the fiber’s optical properties. The four cladding circles radius r_1_ = r_2_ = r_3_ = r_4_ = 50 μm. The design has three pairs of oval shapes. While the outer horizontal two elliptical holes have a width of (a_3_) = 100 μm and height of (b_3_) = 200 μm.

This design achieves efficient detection of chemicals by utilizing evanescent field interactions in air holes, detecting refractive index shifts, absorption, or fluorescence. The elliptical and circular hole design with radial symmetry enhances sensitivity for applications like gas sensing and liquid analysis. Silica glass, conversely, plays a crucial role in achieving high relative sensitivity *RS* while maintaining low confinement loss *L*_*C*_. Key chemicals, Sarin, Soman, and Tabun, are centered on their refractive indices. Silica glass improves robustness and low loss, while the PML boundary condition is 10% of the fiber’s maximum length.

Silica and Zeonex are essential background materials in photonic crystal fiber (PCF) sensors due to their unique optical properties. Silica glass provides high thermal stability, low optical loss, and excellent mechanical strength, making it ideal for high-performance applications. Zeonex, a low-refractive-index polymer, offers flexibility, reduced material loss, and better transmission at specific wavelengths. The sensor with silica glass demonstrated improved sensitivity and reduced confinement loss compared to the Zeonex-based design, enhancing overall performance and efficiency.

**Fig. 2:**
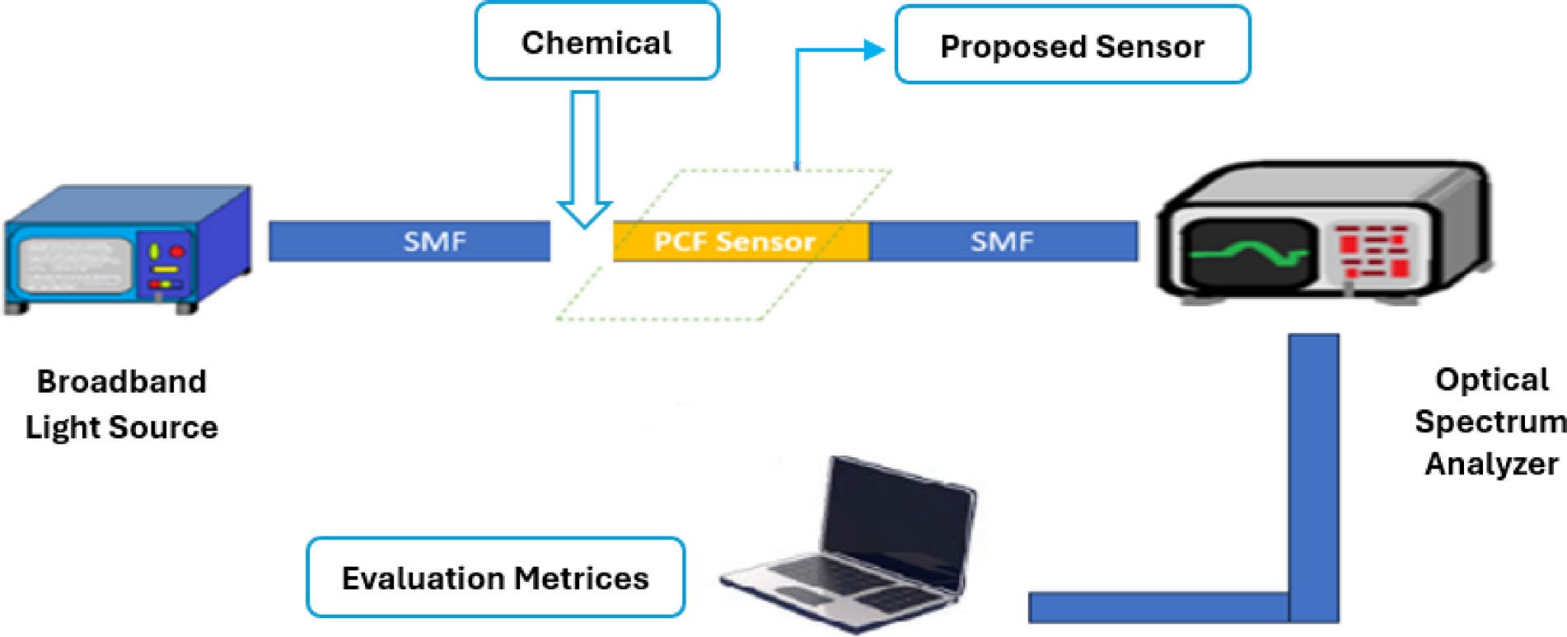
Block diagram of the practical arrangement.

Figure 2 presents the block diagram of the introduced PCF sensor, which utilizes fiber couplers and single-mode fiber (SMF) to analyze incident light. When light propagates through the fiber, an optical spectrum analyzer (OSA) detects variations in light intensity resulting from changes in the refractive index (RI) of chemical molecules. The data acquired by the OSA is subsequently processed by a computer to generate efficiency assessment metrics for different chemical compounds.

The enhanced performance of the proposed sensor is primarily attributed to its innovative structural configuration, which combines an elliptical cladding with a circular core. This design enables improved light confinement and a stronger evanescent field interaction with the surrounding medium, resulting in higher sensitivity and lower signal loss. The elliptical cladding introduces asymmetry that enhances birefringence and optical field distribution, while the circular core ensures stable mode propagation and minimal confinement loss. Unlike earlier designs that primarily relied on Zeonex or Topas as background materials, this sensor leverages silica to further reduce material loss and improve detection reliability. Together, these design choices contribute to a high-performance sensing platform capable of achieving superior optical metrics, such as relative sensitivity up to 99.6% and ultra-low confinement losses, thereby representing a significant advancement in photonic chemical sensing within the THz regime.

While the proposed PCF sensor demonstrates high sensitivity and strong potential for detecting nerve agents such as Sarin, Soman, and Tabun, it is important to recognize the inherent limitations associated with optical detection methods. These include restricted access to the actual toxic agents due to safety regulations, spectral overlap between chemically similar compounds, the challenge of detecting extremely low concentrations, and material cost considerations. By addressing these limitations through careful structural design, the use of safe simulants, and the selection of cost-effective materials like silica, the sensor offers a practical and reliable approach. This balanced understanding of both strengths and constraints reinforces the scientific validity and real-world applicability of the proposed design.

## Analytical mathematics

The effective area (*A*_*eff*_) is a crucial optical property of photonic crystal fiber (PCF). As established, a PCF structure with a larger effective area exhibits higher relative sensitivity (RS). The following equation is utilized to calculate the effective area^11^:

1 $${A_{eff}}=\frac{{{{\left( {\int {\int {\left| E \right|} {}^{2}{d_y}{d_x}} } \right)}^2}}}{{\left( {\int {\int {\left| E \right|} {}^{4}{d_y}{d_x}} } \right)}}~~~~~~~~~~~~{m^2}$$

The fundamental mode propagation necessitates incorporating the electric field distribution (*E*) within the core.

Another critical optical characteristic of the PCF structure is confinement loss (*L*_*C*_). A lower confinement loss enhances relative sensitivity (*RS*), facilitating the efficient detection of chemical substances. The confinement loss (*L*_*C*_) is determined using the following equation^42,43^.:

2 $$Lc=\frac{{4\pi f}}{c}*{I_m}({n_{eff}})~~~~~~~~~~~~dB/m$$

In this context, the speed of photons is denoted as c, while the frequency is represented as f. Additionally, the effective refractive index includes an imaginary component, expressed as *I*_*m*_(*n*_*eff*_).

Relative sensitivity is a crucial optical parameter for detecting chemicals in a PCF structure. Relative sensitivity (*RS*) is determined using the following equation^44^:

3 $$RS=\frac{{{n_r}}}{{{n_e}}} \times E$$

In this equation, *n*_*r*_ and *n*_*e*_ represent the refractive index and effective refractive index, respectively. Additionally, the total interaction between light and matter is calculated through the following expression^44^:

4 $$E=\left( {\frac{{\int_{{sample}} {\operatorname{Re} ({H_y}{E_x} - } {H_y}{E_x}){d_y}{d_x}}}{{\int_{{all}} {\operatorname{Re} ({H_y}{E_x} - {H_y}{E_x}){d_y}{d_x}} }}} \right)*100\%$$

The electric fields in the *x* and *y* directions are represented by *E*_*x*_ and *E*_*y*_​, respectively, while the magnetic fields in the x and y directions are denoted by *H*_*x*_ and *H*_*y*_​.

Effective material loss (*EML*) represents the amount of light that the backdrop material absorbs per unit length, resulting from heat generated by light-matter interactions. A well-designed PCF with minimized background regions reduces EML, calculated through perturbation theory as shown in Eq. (5)^45^:

5 $$EML=\left( {\frac{{\sqrt {\frac{{{\varepsilon _0}}}{{{\mu _0}}}} \int_{{mat}} {{n_{mat}}{{\left| E \right|}^2}{\alpha _{mat}}d} A}}{{\int_{{all}} {0.5*(H*E)\hat {z}dA} }}} \right)~~~~~~~~~~~~c{m^{ - \,1}}$$

In this context, *ε*_*0*_ and *µ*_*0*_ represent the permittivity and permeability of open space, correspondingly. The refractive index of the backdrop substance is denoted by *n*_*mat*_, while *α*_*mat*_ indicates the material’s absorption loss.

The numerical aperture (*NA*) defines the fiber’s ability to accept and transmit incoming light angles. A smaller effective area leads to a higher *NA*, which is beneficial for detection technologies. The *NA* value is calculated using Eq. (6)^11^:

6 $$NA=\frac{1}{{\sqrt {1+\frac{{\pi {f^2}({A_{eff}})}}{{{c^2}}}} }}$$

Birefringence (*B*) quantifies the irregularity in the gap between the core and cladding air holes regions^46^. Disrupting the PCF’s spatial symmetry increases birefringence. The refractive index difference between $\:{{n}_{e}}^{x}$ and $\:{{n}_{e}}^{y}$ determines its value, calculated using Eq. (7)^11^:

7 $$B=\left| {{n_{{e^x}}} - {n_{{e^y}}}} \right|$$

## Results outcomes, and discussion

The proposed sensor methodology is fundamentally based on the precise refractive index (RI) values of various chemical compounds, which are crucial for accurate identification and classification^47^. These RI for Strain, Soman, and Tabun are 1.366, 1.394, and 1.44 respectively. To enhance the sensor’s performance, multiple optimization strategies and experimental procedures have been implemented. The integration of these advanced methodologies can significantly improve sensitivity and overall detection accuracy^48^. Future enhancements to the proposed design could involve integrating advanced computational techniques, particularly deep learning, to further optimize performance and improve the sensor’s detection capabilities^49,50^.

A critical aspect of this study is the optimization of the core radius, which plays a vital role in determining essential assessment metrics such as effective modal area (*A*_*eff*_), numerical aperture (*NA*), confinement loss (*L*_*C*_), equivalent mode volume (*EML*), and relative sensitivity (*RS*). The optimization process was conducted using two different background materials such as Silica and Zeonex, to comprehensively evaluate their influence on sensor performance. Through rigorous computational analysis, the optimal core radius was identified based on a comparative assessment of these materials across all key metrics. Numerical simulations were performed within the frequency range of 1.6 to 3.6 THz using COMSOL Multiphysics to validate the sensor’s performance. The results of these simulations, which highlight the efficiency of the proposed PCF design, are presented in the subsequent sections. This study ultimately aims to develop a highly efficient sensor utilizing an elliptical cladding with a circular core (E-PCF) for the precise detection of hazardous chemical compounds.

### Relative sensitivity (*RS*)

The three chemical compounds’ *RS* fluctuation across the frequency range from 1.6 THz to 3.6 THz. It clearly shows that using Silica glass as a background material gives better response than using Zeonex. Elevating the optical power region of the core region results in a significant increase in RS from 1.6 to 2.6 THz for all compounds, thereby achieving enhanced sensitivity. *RS* demonstrates a modest incremental trend, which is approximately 0.3% in optimal scenarios, from 2.6 THz to 3.6 THz.

**Fig. 3:**
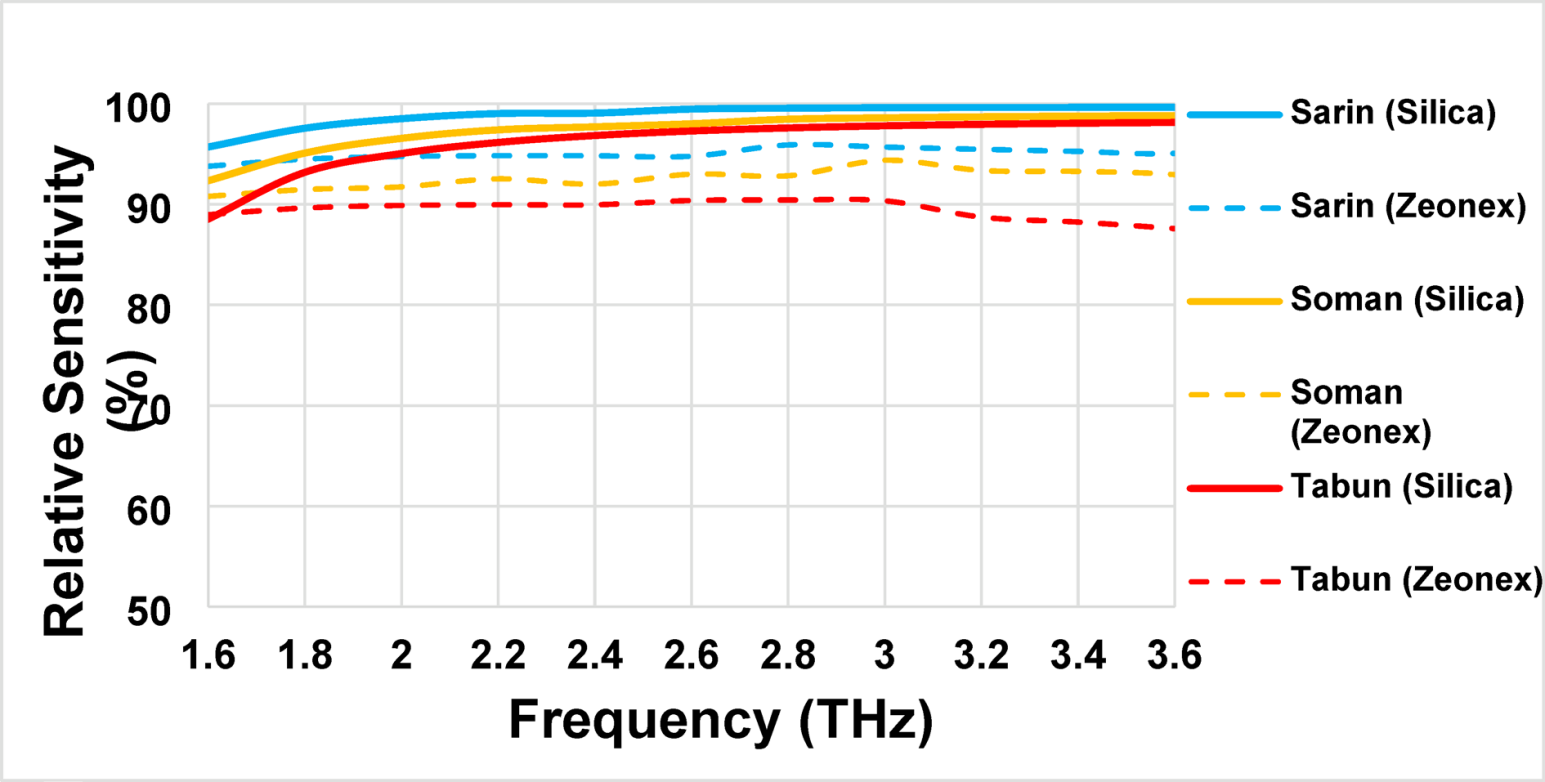
Relative sensitivity (*RS*) of the introduced PCF sensor for detecting Sarin, Soman, and Tabun using Silica and Zeonex across the frequency range of 1.6–3.6 THz.

Figure 3 demonstrates the *RS* of the introduced sensor for sensing three highly toxic chemical compounds—Sarin, Soman, and Tabun—using both Silica and Zeonex as background materials across a frequency range of 1.6 to 3.6 THz. Focusing on the Silica-based design, the sensor exhibits superior *RS* performance for Sarin, achieving values consistently above 96% across the entire frequency range, with a maximum sensitivity approaching 99.6% at frequency from 3.2 to 3.6 THz. For Soman, the *RS* values are slightly lower than Sarin but still maintain high performance, with *RS* ranging from approximately 94% to 98%. In contrast, the detection of Tabun using Silica shows comparatively lower *RS* values, starting around 88% at lower frequencies and peaking near 98.8%. The above outcomes reveal the effectiveness of the Silica-based sensor in achieving high *RS* for detecting Sarin, Soman, and Tabun over the traditional Zeonex based sensor.

### Confinement loss (*L*_*C*_)

The confinement loss (*L*_*C*_) variation for Sarin, Soman, and Tabun across the frequency spectrum of 1.6 THz to 3.6 THz demonstrates that Silica glass, as a background material, provides a superior response compared to Zeonex. Increasing the optical power within the core region leads to a substantial reduction in *L*_*C*_ between 2 THz and 3.6 THz for all three compounds, thereby minimizing loss and enhancing overall sensor performance.

**Fig. 4:**
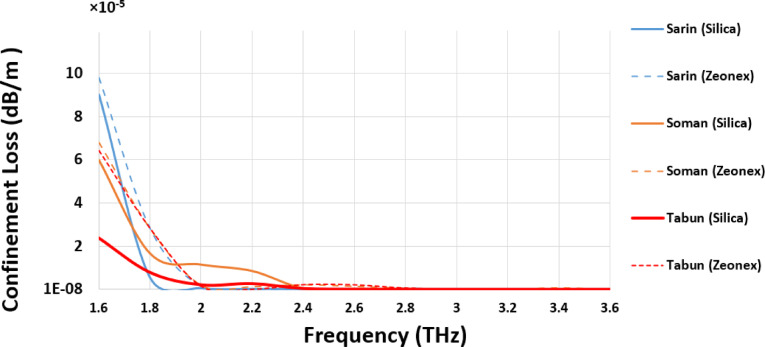
Confinement Loss (*L*_*C*_) of the introduced PCF sensor for detecting Sarin, Soman, and Tabun using Silica and Zeonex across the frequency range of 1.6–3.6 THz.

Figure 4 shows the *L*_*C*_ of the introduced sensor for detecting three highly toxic chemical compounds—Sarin, Soman, and Tabun—using both Silica and Zeonex as background materials across a frequency range of 1.6 to 3.6 THz. Focusing on the Silica-based design, the sensor exhibits lower loss performance for Sarin, achieving values consistently lower than 10⁻^12^ dB/m from 2 to 3.6 THz, while higher loss for low frequencies about 6 × 10⁻^5^ dB/m. For Soman, the *L*_*C*_ values are much like Sarin across a frequency range of 2.4 to 3.6 THz. In contrast, the detection of Tabun using Silica shows slightly higher *L*_*C*_ values of 7 × 10⁻^11^ dB/m across a frequency range of 2.8 to 3.6 THz. The above outcomes reveal the effectiveness of the Silica-based sensor in achieving lower *L*_*C*_ for detecting Sarin, Soman, and Tabun over the traditional Zeonex based sensor.

### Effective material loss (EML)

The effective material loss (*EML*) variation for the three chemical compounds across the frequency range of 1.6 THz to 3.6 THz demonstrates that Silica glass, as a background material, provides superior performance compared to Zeonex. Increasing the optical power concentration within the core region significantly reduces *EML* between 2.6 THz and 3.6 THz for all compounds, leading to improved material loss characteristics.

**Fig. 5:**
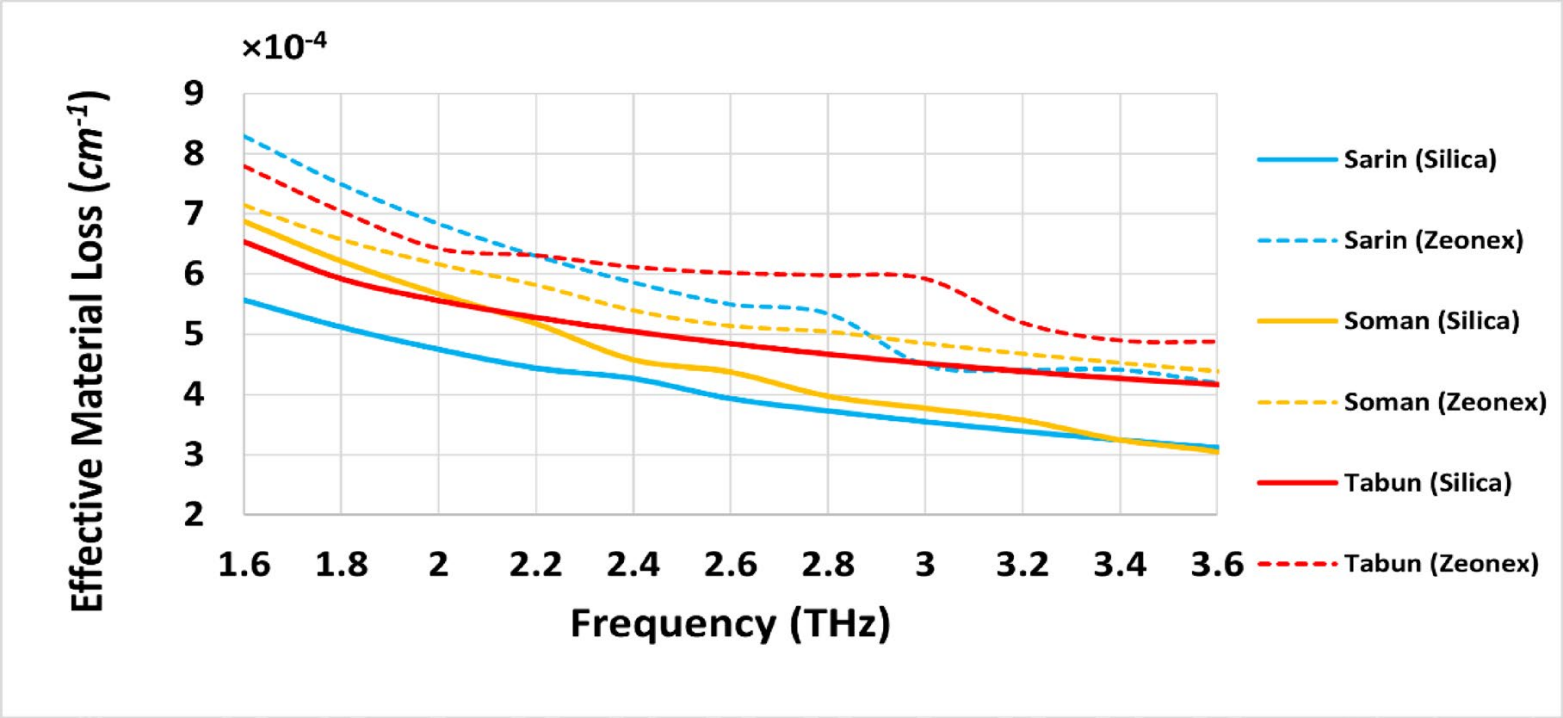
Effective material loss (*EML*) of the introduced PCF sensor for detecting Sarin, Soman, and Tabun using Silica and Zeonex across the frequency range of 1.6–3.6 THz.

Figure 5 shows the *EML* of the introduced sensor for detecting three highly toxic chemical compounds—Sarin, Soman, and Tabun—using both Silica and Zeonex as background materials across a frequency range of 1.6 to 3.6 THz. Focusing on the Silica-based design, the sensor exhibits superior *EML* performance for Sarin, achieving values consistently around 5 × 10^−4^ *cm*^*−1*^ across the entire frequency range, with a minimum *EML* approaching 5 × 10^−4^ *cm*^*−1*^ at frequency from 3 to 3.6 THz. For Soman, the *EML* values are slightly higher than Sarin but still maintain high performance with *EML* of 4.5 × 10^−4^ *cm*^*−1*^. In contrast, the detection of Tabun using Silica shows *EML* of 5 × 10^−4^ *cm*^*−1*^. The above outcomes reveal effectiveness of the Silica-based sensor in achieving better *EML* for detecting Sarin, Soman, and Tabun over the traditional Zeonex based sensor.

### Effective area (*A*_*eff*_)

The effective area (*A*_*eff*_) variation for the three chemical compounds across the frequency range of 1.6 THz to 3.6 THz demonstrates that Silica glass, as a background material, provides superior performance compared to Zeonex. Increasing the optical power concentration within the core region significantly reduces *A*_*eff*_ between 2.8 THz and 3.6 THz for all compounds, thereby enhancing the overall effectiveness of *A*_*eff*_.

**Fig. 6:**
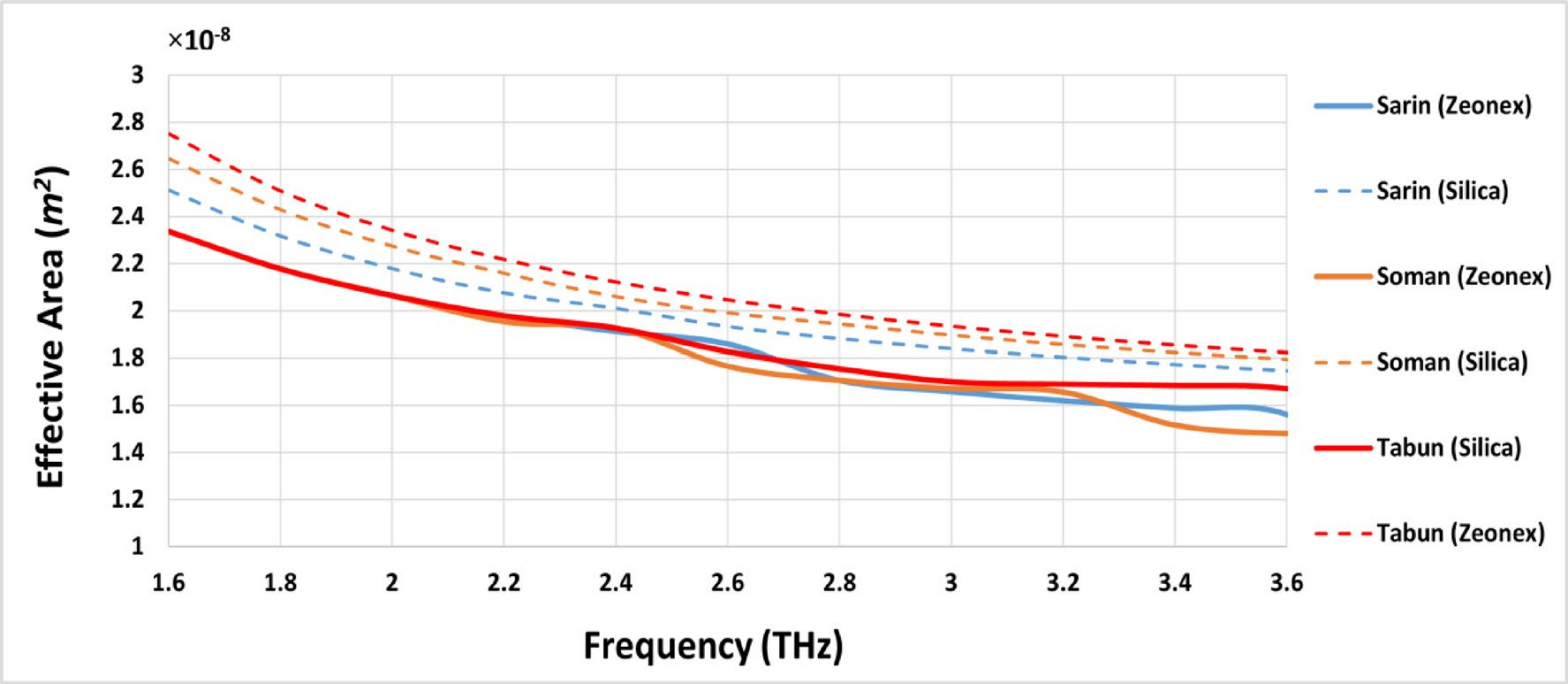
Effective Area (*A*_*eff*_) of the introduced PCF sensor for detecting Sarin, Soman, and Tabun using Silica and Zeonex across the frequency range of 1.6–3.6 THz.

Figure 6 shows the *A*_*eff*_ of the introduced sensor for detecting three highly toxic chemical compounds—Sarin, Soman, and Tabun—using both Silica and Zeonex as background materials across a frequency range of 1.6 to 3.6 THz. Focusing on the Silica-based design, the sensor exhibits superior *A*_*eff*_ performance for Sarin with optimum *A*_*eff*_ of 1.55 × 10^−8^ *m*^*2*^ at frequency from 3 to 3.6 THz. For both Soman and Tabun, the *A*_*eff*_ values are so, much like the Sarin compound. The above outcomes reveal the effectiveness of the Silica-based sensor in providing high *A*_*eff*_ for detecting Sarin, Soman, and Tabun over the traditional Zeonex based sensor.

### Numerical aperture (*NA*)

The numerical aperture (*NA*) specifies the optical fiber’s ability to collect and guide light. A higher *NA* signifies an increased capacity to capture incoming light at broader angles, making it beneficial for PCF-based sensors. However, excessively high *NA* values can lead to multimode propagation, which may be undesirable in certain applications.

**Fig. 7:**
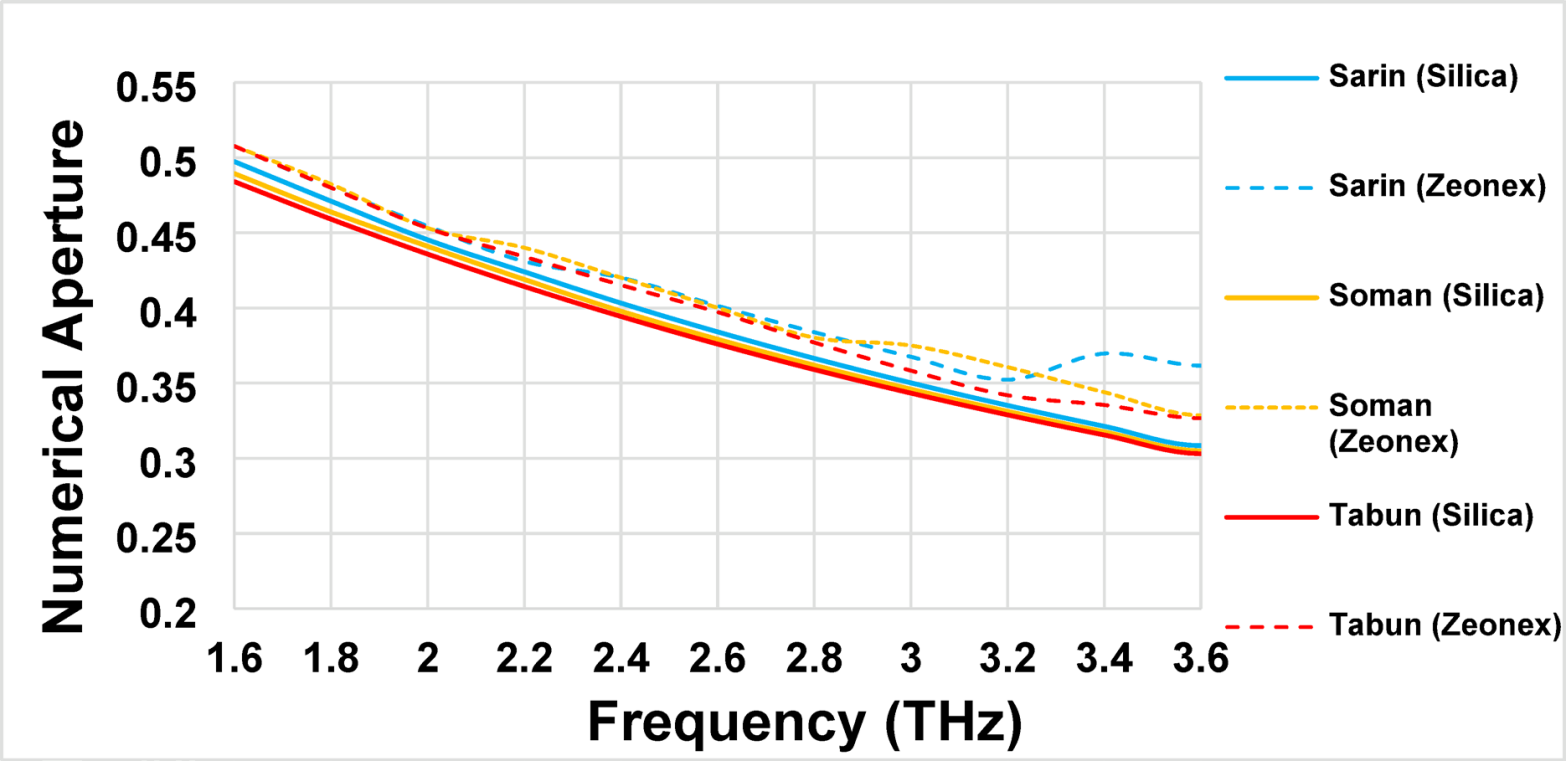
Numerical Aperture (*NA*) of the introduced PCF sensor for detecting Sarin, Soman, and Tabun using Silica and Zeonex across the frequency range of 1.6–3.6 THz.

The variation in *NA* for Sarin, Soman, and Tabun across the frequency spectrum of 1.6 THz to 3.6 THz highlights the superior performance of Silica glass as a background material compared to Zeonex. Figure 5 shows the declining trend of *NA* with increasing frequency. At 1.6 THz, the *NA* values are 0.507, 0.492, and 0.480 for Sarin, Soman, and Tabun, ccorrespondingly. The minimum *NA* values at 3.6 THz are 0.362, 0.329, and 0.327, as shown in Fig. 7. These findings emphasize the efficiency of the Silica-based sensor in achieving enhanced *NA* for detecting these chemical agents compared to Zeonex-based alternatives.

The numerical aperture (*NA*) characterizes an optical fiber’s capacity to capture and transmit light. A higher *NA* indicates an increased ability to accept incoming light at wider angles, making it advantageous for PCF-based sensors. However, an excessively high NA is sometimes associated with multimode fiber behavior, which may not always be desirable.

### Birefringence (*B*)

**Fig. 8:**
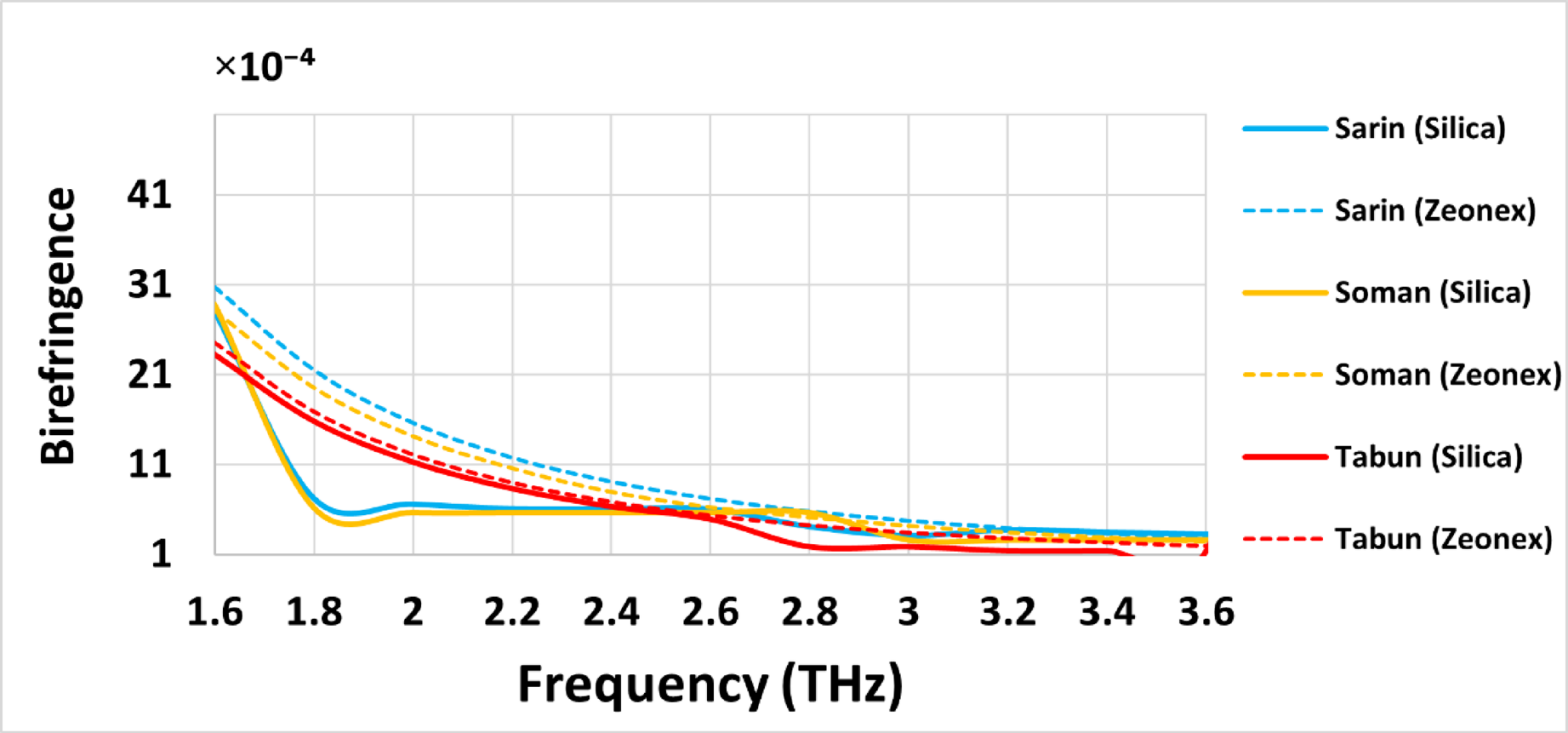
Birefringence (*B*) of the introduced PCF sensor for detecting Sarin, Soman, and Tabun using Silica and Zeonex across the frequency range of 1.6–3.6 THz.

The birefringence (*B*) variation of the three chemical compounds across the 1.6 THz to 3.6 THz frequency range demonstrates that Silica glass, as a background material, provides a superior response compared to Zeonex. Figure 6 shows the birefringence behavior of the designed sensor for detecting highly toxic chemical materials—Sarin, Soman, and Tabun—using Silica and Zeonex as background materials. As frequency increases, the birefringence of the optimized sensor structure decreases, which is expected. A rise in operating frequency reduces the refractive index difference between x- and y-polarization modes, leading to a decline in birefringence. As Shown in Fig. 8, the optimized structure achieves birefringence values of approximately 3.1 × 10⁻⁴, 2.63 × 10⁻⁴, and 1.83 × 10⁻⁴ for Sarin, Soman, and Tabun, respectively, at 3 THz.

Table 1 presents the effect of core radius variation on the sensing performance of the proposed PCF sensor at the optimum frequency of 3.6 THz for Sarin detection. The *RS* increases significantly with core radius, rising from 82.89% at 65 μm to a peak of 98.73% at 80 μm, then slightly decreasing at 85 μm. This trend reflects improved analyte interaction with increasing core size, up to an optimal point. Meanwhile, *L*_*C*_ and *EML* both increase with core radius, particularly beyond 75 μm, indicating that larger cores lead to weaker light confinement and greater absorption. The *A*_*eff*_ also increases steadily, from 1.61 × 10^−8^ m^2^ to 1.97 × 10^−8^ m^2^. These results highlight a trade-off: while *RS* improves with core enlargement, excess increase negatively impacts L_C_ and *EML*, suggesting 75–80 μm as the optimal radius range.

**Table 1:** Optimizing the core radius of the sarin chemical at frequency of 3.6 THz.

Core (µm)	RS (%)	Lc (dB/m)	Aeff (m^2^)	EML (cm^−1^)
65	82.89	1.54 × 10^−11^	1.61 × 10^−8^	1.22 × 10^−4^
70	90.95	1.75 × 10^−11^	1.74 × 10^−8^	2.78 × 10^−4^
75	98.13	1.89 × 10^−11^	1.82 × 10^−8^	3.12 × 10^−4^
80	98.73	2.66 × 10^−10^	1.82 × 10^−8^	3.40 × 10^−4^
85	98.12	9.93 × 10^−10^	1.97 × 10^−8^	4.56 × 10^−4^

Table 2 illustrates the effect of temperature on the sensing parameters of the proposed optical PCF sensor at the optimum frequency of 3.6 THz for Sarin detection. As temperature increases from 200 K to 400 K, *L*_*C*_ and *EML* show a clear increasing trend, indicating that higher thermal energy leads to reduced mode confinement and enhanced material absorption. Specifically, *L*_*C*_ rises from 1.35 × 10^–11^ dB/m at 200 K to 2.92 × 10^–11^ dB/m at 400 K, while *EML* increases from 2.53 × 10^−4^ cm^−1^ at 200 K to 3.35 × 10^−4^ cm^−1^ at 400 K. In contrast, the *RS* shows a slight decreasing trend, dropping from 98.79703% at 200 K to 98.77659% at 400 K, although the variation remains minimal. The *A*_*eff*​_ remains nearly constant throughout, indicating thermal stability in the fiber geometry. These results highlight the importance of thermal effects in sensor accuracy and performance under varying environmental conditions.

**Table 2:** Temperature effect of the sarin chemical at optimal frequency of 3.6 THz.

Temperature (K)	RS (%)	Lc (dB/m)	A_eff_ (m^2^)	EML (cm^−1^)
200	98.79703	1.35 × 10^–11^	1.80 × 10^−8^	2.53 × 10^−4^
250	98.67546	1.65 × 10^–11^	1.82 × 10^−8^	2.72 × 10^−3^
275	98.67264	1.85 × 10^–11^	1.82 × 10^−8^	3.02 × 10^−3^
297 (Room Temp.)	98.78634	1.89 × 10^–11^	1.82 × 10^−8^	3.12 × 10^−4^
325	98.78376	2.09 × 10^–11^	1.82 × 10^−8^	3.18 × 10^−4^
350	98.78138	2.32 × 10^–11^	1.82 × 10^−8^	3.24 × 10^−4^
400	98.77659	2.92 × 10^–11^	1.82 × 10^−8^	3.35 × 10^−4^

Table 3 evaluates the impact of a ± 2% fabrication tolerance on the sensing parameters of the proposed PCF sensor at the optimum frequency of 3.6 THz for Sarin detection. The *RS* remains stable, ranging from 98.01% to 98.59%, confirming the sensor’s robustness against minor geometric deviations. *L*_*C*_ shows a nonlinear response: it decreases to 1.08 × 10^−11^ dB/m for a + 2% change but increases to 2.11 × 10^−11^ dB/m at −2%, indicating that slight expansion improves mode confinement. The *A*_*eff*_ remains nearly constant. However, *EML* increases significantly with geometric deviation, rising from 3.12 × 10^−4^ cm⁻¹ (proposed) to 4.48 × 10^−4^ cm⁻¹ (+ 2%) and 6.77 × 10^−4^ cm⁻¹ (−2%). These results highlight the importance of precision in fabrication, particularly to minimize *EML* and maintain optimal detection efficiency.

**Table 3:** Fabrication tolerance of the proposed design for sarin chemical at optimal frequency of 3.6 THz.

Design Geometry	RS (%)	Lc (dB/m)	A_eff_ (m^2^)	EML (cm^−1^)
−2%	98.01	2.11 × 10^–11^	1.83 × 10^−8^	6.77 × 10^−4^
**Proposed**	98.13	1.89 × 10^–11^	1.82 × 10^−8^	3.12 × 10^−4^
+ 2%	98.59	1.08 × 10^–11^	1.82 × 10^−8^	4.48 × 10^−4^

## Comparative analysis

Comparative study is essential to evaluate the performance of the introduced PCF sensor against recently developed designs. This section systematically examines key factors like *RS*, *L*_*C*_, and *EML*, highlighting the advantages of the proposed sensor in achieving superior efficiency for THz chemical detection.

Table 4 presents a comparison of the inrtoduced elliptical cladding circular-core PCF sensor with recently published PCF-based sensors for the detection of Sarin, Soman, and Tabun compounds in the THz regime. The elliptical cladding design with a circular core enhances light confinement and stability, improving overall sensor efficiency. Material Selection Impact: Unlike previous works that used Topas or Zeonex, the use of Silica contributes to higher stability and lower loss, making the sensor more practical for real-world applications. From the table, it is evident that the proposed sensor outperforms previous designs in several key aspects.

At an operating frequency range of 1.6–3.6 THz, the proposed sensor achieves higher relative sensitivity (99.6%–98.8%), compared to a maximum of 98.5% in^51^ and 96.12% in^52^. The *EML* of 4.5 × 10⁻⁴ cm⁻¹ is significantly reduced compared to other designs, ensuring low optical losses. The confinement loss (3.6 × 10⁻¹³ cm⁻¹) is also very low, indicating strong light guidance in the core. Unlike previous designs that used Topas or Zeonex as background material, the proposed sensor uses Silica, which contributes to better stability and lower loss characteristics. The numerical aperture of 0.507–0.327 suggests a wide range of light acceptance, which is beneficial for efficient light propagation and detection performance. The PCF sensor described in^53^ demonstrates a low confinement loss of 1.7 × 10⁻¹⁴ cm⁻¹; however, it exhibits lower sensitivity and higher effective material loss (*EML*) compared to the proposed sensor. In^51^, the design utilizing a rectangular hollow core for detecting Sarin, Soman, and Tabun achieves an optimal confinement loss of 10⁻¹⁶ dB/m. However, this design suffers from a higher EML than the proposed sensor. In ref^54^., the authors introduced a PCF-based sensor with a confinement loss of approximately 8.34 × 10⁻¹³ cm⁻¹, but it also displays lower sensitivity and higher *EML* than the proposed design. optimal birefringence efficiency, achieving a value of 10^−5^ achieved through the proposed PCF. Finally, the numerical aperture (0.507–0.327) indicates a broad range of light acceptance, making the sensor highly adaptable for THz applications.

While the studies in Ref^55–58^. focused on detecting substances such as petrochemicals, water, milk, and amino acids, the proposed sensor is uniquely designed to target highly toxic nerve agents namely Sarin, Soman, and Tabun within an operating frequency range of 1.6–3.6 THz. It achieves an exceptional relative sensitivity of 99.6%, the highest among all designs compared, surpassing the 99.18% reported in Ref^58^. Furthermore, the sensor demonstrates a low L_C_ of 3.6 × 10^–13^ dB/m and a moderate EML of 4.5 × 10^−4^ cm^−1^, reflecting an optimal balance between detection sensitivity and signal attenuation. The design also yields a notable birefringence of 1.83 × 10⁻⁴, which is either absent or negligible in earlier works. Additionally, the wide numerical aperture (0.507–0.327) enhances optical coupling efficiency. Collectively, these features underscore the proposed elliptical-cladding circular-core PCF sensor’s superior capability for detecting critical airborne chemical threats. Finally, the numerical aperture (0.507–0.327) indicates a broad range of light acceptance, making the sensor highly adaptable for THz applications.

**Table 4:** A comparative analysis between the introduced sensor and recently published PCF sensors operating in the Terahertz (THz) spectrum.

Ref	Lattice type	Detection target	Operating frequency (THz)	Background Material	Effective area (m^2^)	Relative Sensitivity (%)	Birefringence	Confinement loss (dB/m)	EML (cm^−1^)	Numerical aperture
^53^ 2020	Hollow Core (Rectangular)	Sarin, Soman and Tabun	1–2	Zeonex	ـــــ	94.4	0.00682	1.71 × 10^−14^ cm^−1^	0.00859	ـــــ
^59^ 2023	Circular Core	Sarin, Soman and Tabun	0.6–1.8	Zeonex	ـــــ	94	ـــــ	10^−14^ dB/m	ـــــ	ـــــ
^52^ 2021	Rectangular Core	Sarin, Soman and Tabun	1–3	Topas	1.640 × 10^5^ µm^2^	96.12	ـــــ	3.58 × 10^−13^ cm^−1^	0.0097	ـــــ
^51^ 2023	Hollow Core (Circular)	Urine	1.5–5	Topas	5.06 × 10^−8^	98.5	ـــــ	10^−16^ cm^−1^	0.0173	ـــــ
^54^ 2022	Hollow Core (Square)	Chloropicrin, Ethyl-bromide	1.2	polymer Zeonex	1.56 × 10^5^ µm^2^	94.6	ـــــ	8.34 × 10^–13^ cm^−1^	0.007	0.2203
^55^ 2023	Square Core	Petrochemicals	1.2–3.8	Zeonex	90,600 µm^2^	97	ـــــ	1.32 × 10^−14^ cm^−1^	0.0105	ـــــ
^56^ 2022	Circular Hollow Core	Water	0.5–1.5	Fused silica, BK7 glass	8.1 × 10^5^ µm^2^	92	ـــــ	8.2 × 10^−16^ cm^−1^	2.4 × 10^−4^	ـــــ
^57^ 2022	Rectangular Core	Milk	1–3	Zeonex	18,7870 µm^2^	94.75	0.0044	ـــــ	0.00799	ـــــ
^58^ 2024	Hollow Rectangular Core	Amino acids	1.5–3.5	Zeonex	1.41 × 10^5^ µm^2^	99.18	ـــــ	1.04 × 10^–14^ cm^−1^	0.0063	0.1353
^60^ 2021	Hexahedron Core	Ethanol, Benzene, and Water	1–3	Zeonex	1.32 × 10^−7^	88.36	ـــــ	5.55 × 10^–08^	0.00699	ـــــ
Prop. sensor	Elliptical cladding with Circular Core	Sarin, Soman and Tabun	1.6–3.6	Silica	1.55 × 10^−8^	99.6	1.83 × 10^−4^	3.6 × 10⁻^13^	4.5 × 10^−4^	0.507 − 0.327

## Conclusions

A novel elliptical cladding photonic crystal fiber (E-PCF) with a circular core has been designed for terahertz (THz) waveguides to detect highly toxic chemical agents such as Sarin, Soman, and Tabun while analyzing graphical data. Silica glass and Zeonex are employed as background materials, with silica glass exhibiting superior sensitivity and lower confinement loss compared to Zeonex. Using the COMSOL Multiphysics finite element method (FEM), the proposed sensor achieves high relative sensitivities of 99.6%, 98.8%, and 98%, along with minimal confinement losses of 3.6 × 10⁻^13^ dB/m, 1.1 × 10⁻¹² dB/m, and 7.6 × 10⁻^11^ dB/m for Sarin, Soman, and Tabun, respectively. This study explores key parameters, including effective area, mode index, and power fraction in core air holes. The proposed photonic crystal fiber demonstrates significant potential for detecting these hazardous substances, with applications in biomedical research, industrial quality control, material analysis, gas sensing, micro-optics, and clinical diagnostics. Its high sensitivity and low loss make it a crucial advancement in THz technology for chemical sensing and safety monitoring.

## Data Availability

The datasets used and/or analysed during the current study are available from the corresponding author (*Dr. Omar E. Khedr*) on reasonable request.
